# Design and Synthesis of ^99m^TcN-Labeled Dextran-Mannose Derivatives for Sentinel Lymph Node Detection

**DOI:** 10.3390/ph11030070

**Published:** 2018-07-16

**Authors:** Alessandra Boschi, Micòl Pasquali, Claudio Trapella, Alessandro Massi, Petra Martini, Adriano Duatti, Remo Guerrini, Vinicio Zanirato, Anna Fantinati, Erika Marzola, Melchiore Giganti, Licia Uccelli

**Affiliations:** 1 Department of Morphology, Surgery and Experimental Medicine, University of Ferrara, Ferrara 44121, Italy; petra.martini@unife.it (P.M.); melchiore.giganti@unife.it (M.G.); licia.uccelli@unife.it (L.U.); 2 Department of Physic and Earth Science, University of Ferrara, Ferrara 44122, Italy; micol.pasquali@unife.it; 3 Department of Chemical and Pharmaceutical Sciences, University of Ferrara, Ferrara 44121, Italy; claudio.trapella@unife.it (C.T.); alessandro.massi@unife.it (A.M.); adriano.duatti@unife.it (A.D.); remo.guerrini@unife.it (R.G.); vinicio.zanirato@unife.it (V.Z.); anna.fantinati@unife.it (A.F.); erika.marzola@unife.it (E.M.)

**Keywords:** sentinel lymph node, dextran, mannose, ^99m^Tc-radiopharmaceuticals

## Abstract

Background: New approaches based on the receptor-targeted molecular interaction have been recently developed with the aim to investigate specific probes for sentinel lymph nodes. In particular, the mannose receptors expressed by lymph node macrophages became an attractive target and different multifunctional mannose derivate ligands for the labeling with ^99m^Tc have been developed. In this study, we report the synthesis of a specific class of dextran-based, macromolecular, multifunctional ligands specially designed for labeling with the highly stable [^99m^Tc≡N]^2+^ core. Methods: The ligands have been obtained by appending to a macromolecular dextran scaffold pendant arms bearing a chelating moiety for the metallic group and a mannosyl residue for allowing the interaction of the resulting macromolecular ^99m^Tc conjugate with specific receptors on the external membrane of macrophages. Two different chelating systems have been selected, S-methyl dithiocarbazate [H_2_N‒NH‒C(=S)SCH_3_=HDTCZ] and a sequence of two cysteine residues, that in combination with a monophosphine coligand, are able to bind the [^99m^Tc≡N]^2+^ core. Conclusions: High-specific-activity labeling has been obtained by simple mixing and heating of the [^99m^Tc≡N]^2+^ group with the new mannose-dextran derivatives.

## 1. Introduction

The sentinel lymph node (SLN) is defined as the first lymph node that receives lymphatic drainage as well as metastatic cells from the primary tumor sites. An accurate identification and characterization of SLNs is very important as it helps the physician to decide the extension of surgery, the tumor staging, and the development of an appropriate treatment plan.

Sentinel lymph node detection (SLND) is a radionuclide-based technique for imaging regional lymph node drainage systems, performed by injecting small radiolabeled particles (20 to 500 nm). This technique has become the standard of care in breast cancer [1,2] and melanoma [3,4], and is increasingly being applied to other solid cancers with high metastatic potential in lymph nodes, such as oral and oropharyngeal squamous cell carcinoma [5].

The most frequently used radiopharmaceuticals for SLND are ^99m^Tc-labelled colloidal particles. However, they are characterized by nonideal properties [6,7,8]; in particular, their uptake mechanism is driven by passive diffusion and show slow clearance rate from the injection site or low residence time in the SLN [9,10,11,12]. From the clinical point of view, an ideal tracer must combine persistent retention in the SLN, low distal lymph node accumulation, fast clearance rate from the injection site, safe radiation exposure level, and lack of toxicity.

New approaches based on the receptor-targeted molecular interaction have been developed with the aim to investigate specific probes for SLN. In particular, the mannose receptors expressed by lymph node macrophages became an attractive target [13,14], and multifunctional mannose-derivate ligands have been studied for the labeling with ^99m^Tc [8,11,12]. A multifunctional ligand is commonly depicted as a molecular moiety being sufficiently large in size to accommodate a number of different chemical groups performing specific chemical and biological functions. Dextran provides a convenient macromolecular scaffold for hosting a relatively large number of functional groups and it has been recently employed to develop the ^99m^Tc radiopharmaceutical (Lymphoseek^®^) for SLND [15]. The basic design of this new agent involves appending at various positions of the dextran structure a number of diethylenetriaminepentaacetic acid (DTPA) groups for the chelation of the metal together with a number of mannose residues for recognition by specific receptors on the macrophage’s membrane. A strong limitation of this approach comes from the fact that DTPA is not considered an optimal chelating system for ^99m^Tc and, as a consequence, the stability of the resulting conjugate macromolecular complex is poor. Furthermore, the technetium chemistry with DTPA is not well defined and some controversy about the nature of the complex formed with this metal exists [16]. Recently, aiming to provide more stable and chemically well-defined target-specific ^99m^Tc complexes for SLND, ^99m^Tc-tricarbonyl technology has been applied to label mannosylated-dextran conjugates in combination with pyrazolyldiamine chelator that selectively react with the *fac*-[^99m^Tc(CO)_3_(H_2_O)_3_]^+^ metal fragment [11]. According to the mannosylated-dextran conjugates strategy, ‘4 + 1’ mannosylated-dextran Tc(III) mixed-ligand complexes have been also reported [17].

Based on the dextran derivatives functionalized with mannose units’ principle, we report in this work the design, the synthesis, and the characterization of a specific class of dextran-mannose multifunctional ligands specially designed for binding to [^99m^TcN]^2+^ group. The coordination chemistry of this metallic synthon is very well established [18,19,20] and can be efficiently manipulated by a careful selection of the coordinating atoms type bound to the [^99m^TcN]^2+^ group.

The compound S-methyl dithiocarbazate [H_2_N‒NH‒C(=S)SCH_3_=HDTCZ] has been selected as the first chelating system to be investigated in the production of a multifunctional ligand for SLND based on ^99m^Tc nitrido chemistry. DTCZ strongly binds to the [^99m^Tc≡N]^2+^ core, through the neutral thiocarbonyl sulfur atom and the deprotonated terminal amine nitrogen atom, forming both mono- and bis-substituted complexes [21,22]. Thus, the synthesis of this ligand is described in the following sections.

**Figure 1:**
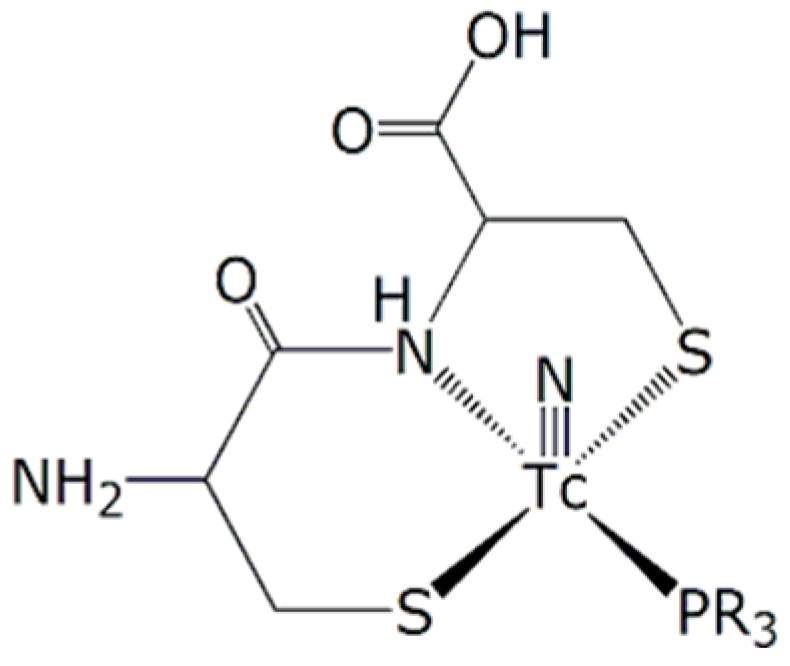
Structure of ‘3 + 1’ ^99m^Tc nitrido complexes with the Cys-Cys chelating system.

Another convenient chelating system for the [^99m^Tc≡N]^2+^ core is provided by the so-called ‘3 + 1’ method. This approach stems from the finding that the coordination arrangement composed by a tridentate π-donor ligand, having [S^−^, N, S^−^] as a set of donor atoms, and a monodentate π-acceptor monophosphine ligand (PR_3_) usually exhibits a high stability when bound to a [^99m^Tc≡N]^2+^ group in a square pyramidal geometry. A very convenient tridentate [S^−^, N, S^−^] chelating system is provided by the simple combinations of two terminal cysteine aminoacids (Cys-Cys). The schematic structure of of ‘3 + 1’ ^99m^Tc nitrido complexes with the Cys-Cys chelating system is illustrated in Figure 1.

In the following, the preparation and stability studies of ^99m^Tc-radiopharmaceuticals containing mannose-dextran derivatives are described.

## 2. Results and Discussion

### 2.1. Design of a Dextran-Mannose Multifunctional Ligand for Coordination to the [^99m^Tc≡N]^2+^ Core

**Figure 2:**
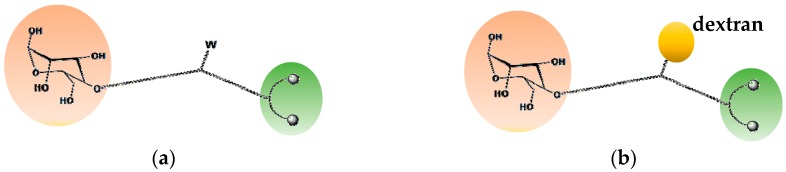
Schematic drawing of a multifunctional fragment with a reactive group (W) (**a**) for binding to dextran (**b**).

A chemical approach, usually employed for attaching different functional groups to a dextran scaffold, consists of hanging them at different positions of the polymeric chain. We used here a simplified strategy that allowed a more careful control of the number of functional groups introduced into the final macromolecule. This approach is schematically illustrated in Figure 2. As mentioned above, two functional groups are required for obtaining a new ^99m^Tc nitrido agent for SLND, namely a suitable chelating group and a mannosyl residue. Each group was placed at one terminus of a linear chain of atoms that was also equipped with a reactive group (W) in its central position (Figure 2a). In turn, this latter group was reacted with another suitable reactive moiety, previously attached to the dextran scaffold, thus forming a stable linkage (click chemistry) (Figure 2b). Through this reaction, both functionalities remained strongly tethered to the macromolecular backbone as branched pendant arms.

### 2.2. Synthesis of Dextran-Mannosyl Multifunctional Ligands for the [^99m^Tc≡N]^2+^ Core

**Figure 3:**
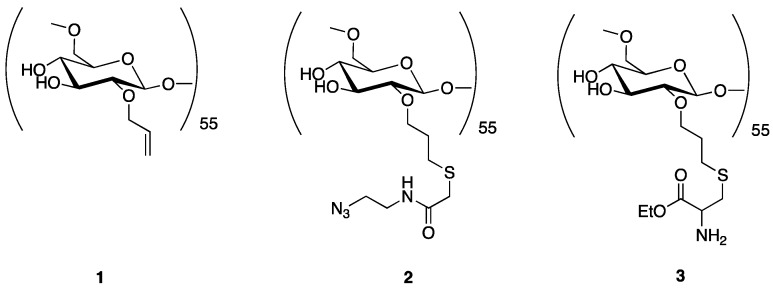
Dextran derivatives used in the synthesis of the new dextran-mannose multifunctional ligands.

As mentioned above, the overall synthetic strategy employed here involved the preliminary preparation of a linear trifunctional fragment bearing a mannose residue at one terminus, a chelating group for the [^99m^Tc≡N]^2+^ core at the other terminus, and a reactive alkyne or sulfide group placed almost in the center of the linear chain (W in Figure 2a). These fragments were subsequently linked to the dextran backbone using three different procedures: (a) Thiol-ene chemistry [23]; (b) azide-alkyne Huysgen cycloaddition (click chemistry) [24], and (c) amide condensation. Specifically, the allyl-dextran (**1**), the azido-dextran (**2**), and cysteine-dextran derivative (**3**) (Figure 3) were used with thiolene condensation, click chemistry, and amide bond formation, respectively.

#### 2.2.1. Synthesis of Dextran Derivatives

**Figure 4:**
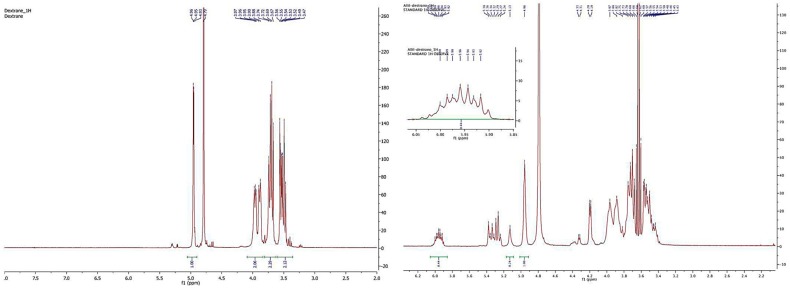
^1^H-NMR spectra of dextran (**left**) and allyl dextran (**right**).

The commercially available dextran with average molecular weight 10,000 Da was reacted with allyl bromide, sodium hydroxide, and sodium borohydride to obtain the dextran derivative **1**. The product was purified by repeated precipitation with ethanol and dried under vacuum to achieve a constant weight. The final loading of dextran polymer was achieved by comparison of ^1^H-NMR spectra. In particular, the chemical shift and the integral value of anomeric protons (nonsubstituted and substituted dextran) at 4.96 ppm and 5.13 ppm were reported to the allylic proton at 5.96 ppm. The loading was about 18 allylic groups for every polymeric unit (18 allyl moiety for 55 monomeric sugar in the dextran); see Figure 4. As shown by the NMR spectra of allyl dextran, only one compound was reported. As suggested by Pirmettis and coworkers [12], and in agreement with our analytical data, position 2 of dextran was the most accessible, probably for steric reasons. In our experience, no other positions were touched by the alkyl group using allyl bromide and sodium hydroxide as reagents.

**Figure 5:**
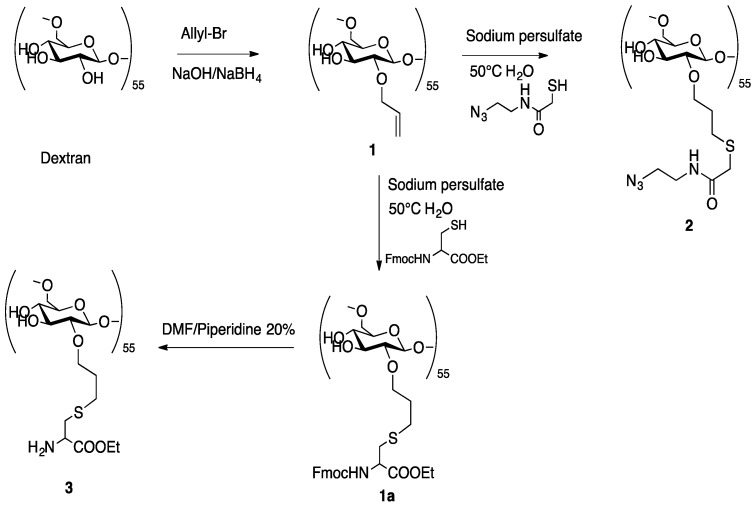
Synthesis of dextran derivatives.

Compound **1** was then treated with the azido-thioglicol amide at 50 °C in the presence of ammonium persulfate to obtain the corresponding thiol-ene adduct **2** (Figure 5). The same procedure was adopted for the reaction with the Fmoc-cysteine ethyl ester to obtain compound **1a** that was directly deprotected to obtain the free amine compound **3**.

#### 2.2.2. Synthesis of a Dextran-DTCZ Multifunctional Ligand

**Figure 6:**
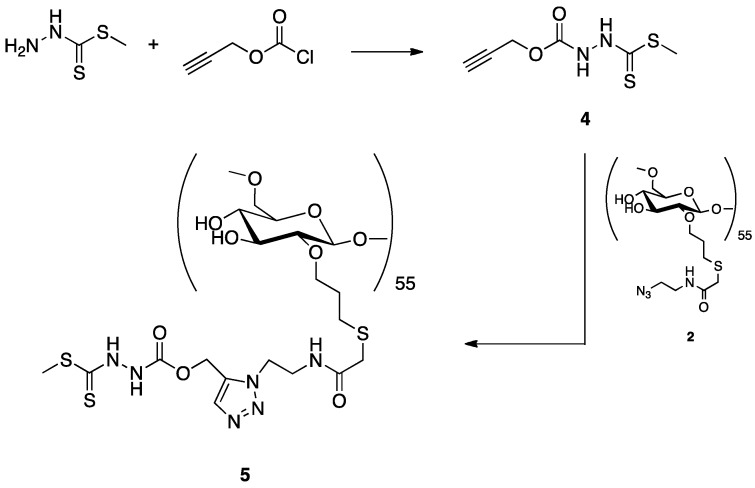
Schematic drawing of the synthesis of dextran-DTCZ (**5**).

A preliminary synthesis of a dextran-DTCZ derivative was carried out according to the reaction scheme depicted in Figure 6. HDTCZ was firstly functionalized with an alkyne moiety (**4**) and then linked to azido-dextran (**2**) by click chemistry. Preliminary labeling of the resulting ligand (**5**) was carried out in physiological solution by simple mixing with the [^99m^Tc*≡*N]^2+^ intermediate prepared by reaction of [^99m^Tc][TcO_4_]^−^ with succinic dihydrazide (SDH) in the presence of Sn^2+^ ions. Although the labeling yield was >90%, it was found that **5** was highly unstable also in the solid state. In particular, after freeze-drying, the labelling yield dropped to 50%, thus indicating that the DTCZ group was partially removed from the dextran scaffold by the lyophilization process. Because of these difficulties, this type of dextran derivative for SLND was abandoned and the mechanism of DTCZ decomposition has not been deepened.

### 2.3. Synthesis of 2-(2,3,4,6-tetra-O-acetyl-β-d-mannopyranosyl)-Acetic Acid

**Figure 7:**
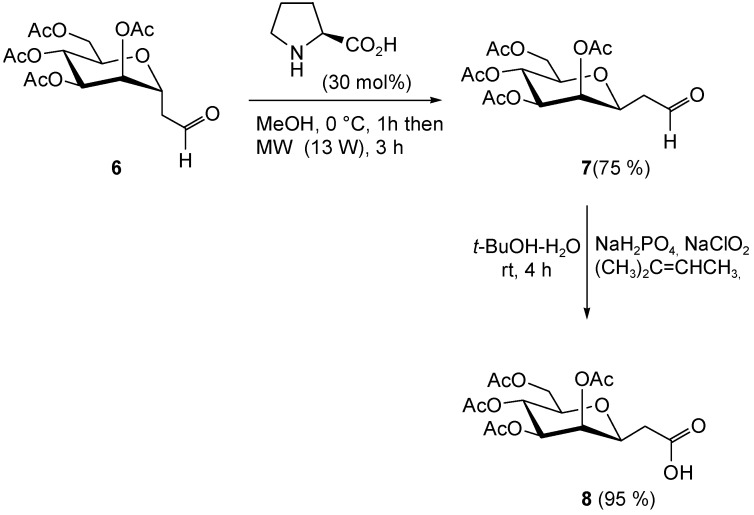
Synthesis of 2-(2,3,4,6-tetra-*O*-acetyl-β-d-mannopyranosyl)-acetic acid **8**.

The novel 2-(2,3,4,6-tetra-*O*-acetyl-β-d-mannopyranosyl)-acetic acid **8** was synthesized by oxidation of the corresponding β-d-mannopyranosyl acetaldehyde **7**, which in turn was prepared from the isomeric α-aldehyde **6 [25]** by a previously optimized anomerization procedure [26]. It is worth noting that the challenging mannosyl derivative **8**, that is a β-C-mannoside, was suitably designed to display a metabolically stable carbon–carbon anomeric linkage between the sugar moiety and the carboxylic functionality, thus preventing the corresponding glycoconjugates from chemical and enzymatic degradation (deglycosylation) in vivo [27]. Accordingly, the α-mannosyl acetaldehyde **6** was dissolved in MeOH and treated with l-proline organocatalyst (30 mol%), which promoted the anomeric process to the corresponding β-aldehyde **7** with the aid of microwave (MW) dielectric heating (constant power at 13 W for 3 h). The target β-mannosyl aldehyde **2** (thermodynamic product) was duly isolated in pure form by column chromatography (75% yield) and then subjected to a standard oxidation procedure with sodium chlorite [28] to give the corresponding acid **8** in almost quantitative yield (Figure 7).

### 2.4. Synthesis of a Dextran-Mannose-CysCys Multifunctional Ligand

**Figure 8:**
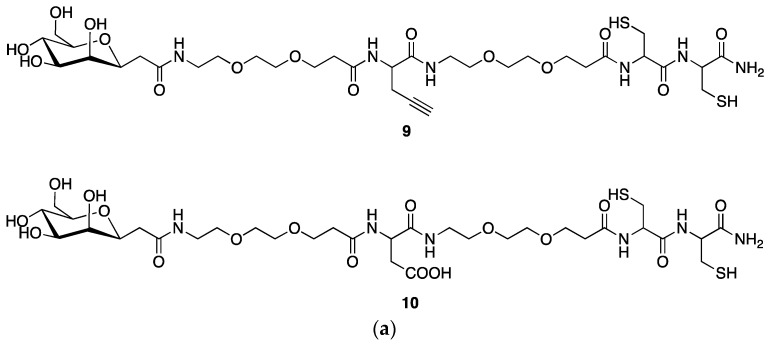

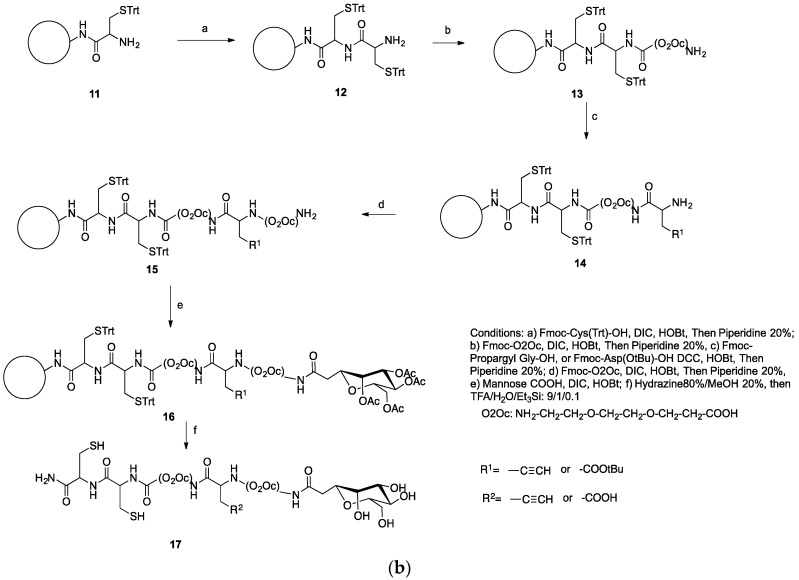
Structure (**a**) and synthesis (**b**) of mannosyl-CysCys ligands.

A pair of multifunctional mannosylated CysCys ligands **9** and **10**, (Figure 8a) suitable for the labeling through the 3 + 1 method, was obtained using the reactions depicted in Figure 8b.

**Figure 9:**
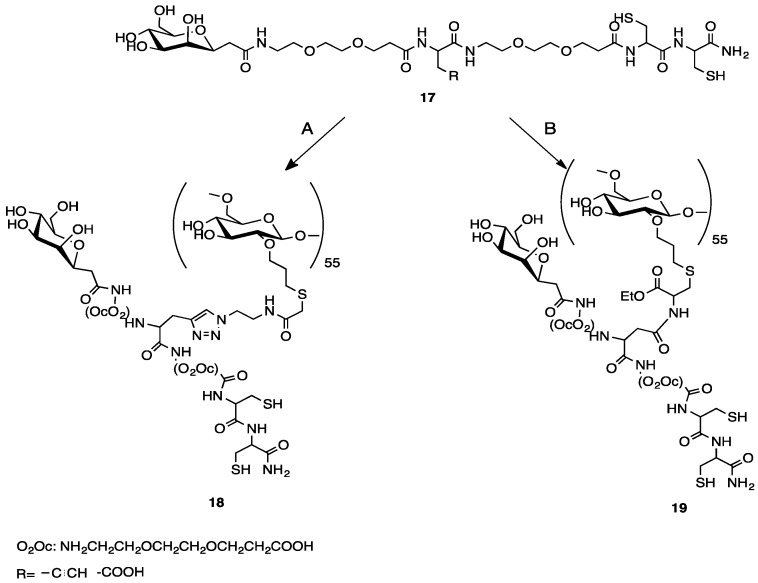
Synthesis and structure of dextran-mannosyl-CysCys ligands **18** and **19**.

As a first step, the two linear pseudopeptides **9** and **10** were produced following the procedures illustrated in Figure 8b. Essentially, these compounds have the same basic structural features given by a terminal mannose group and a terminal combination of two cysteine aminoacids, but differ from the reactive group positioned approximately at the center of the linear pseudopeptide chain. In particular, **9** carries an alkyne group that is replaced by a carboxylic group in **10**. Pseudopeptide **9** was then linked to the dextran derivatives **2** through click chemistry reaction. Instead, pseudopeptide **10** was appended to the dextran derivative **3** via amide condensation (Figure 9).

### 2.5. Preparation of ^99m^TcN-“3 + 1” Labeled Dextran-Mannose Derivates

**Figure 10:**
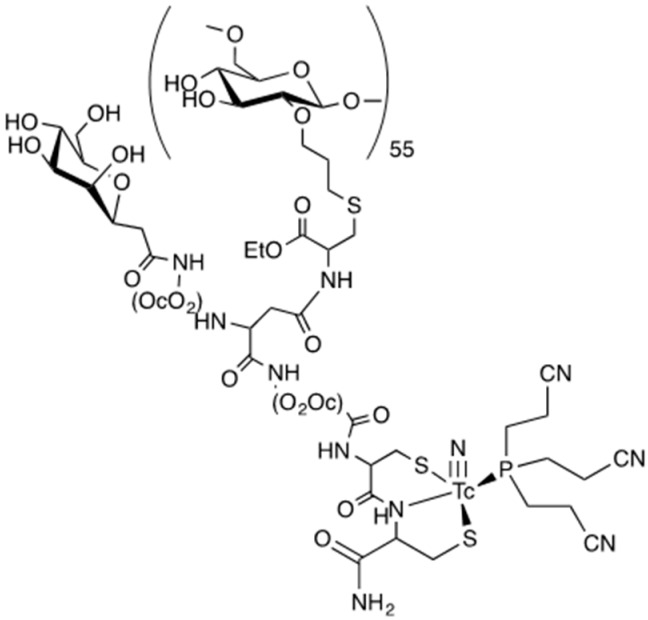
Representative labeling of the dextran-mannosyl-CysCys ligand **19** with the [^99m^Tc≡N]^2+^ core.

**Figure 11:**
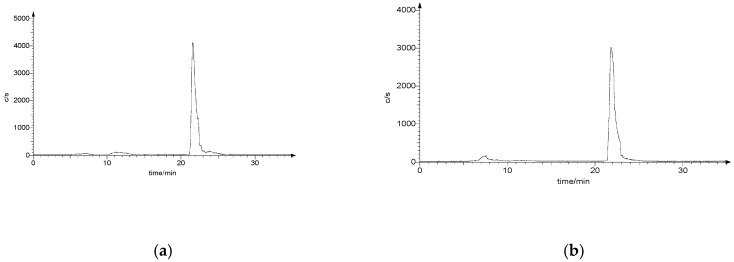
HPLC chromatograms of [^99m^Tc≡N(**18**)PCN] (**a**) and [^99m^Tc≡N(**19**)PCN] (**b**) complexes.

The resulting multifunctional ligands **18** and **19** (Figure 9) were labeled with the [^99m^Tc≡N]^2+^ core by applying the 3 + 1 approach, as shown in Figure 10. The monophosphine PCN (tris-cyanoethyl phosphane) was employed as ancillary ligand. Labeling yields were >95% (Figure 11a,b), and the resulting complexes exhibited a prolonged stability (>6 h) in physiological solution.

The formulation developed to prepare the ^99m^TcN-“3+1” labeled dextran-mannose compound contains 0.1 mg of dextran-derivate, about half of that involved in the Lymphoseek^®^ formulation (0.250 mg). Therefore, even if this dextran-derivate could have hypersensitivity effect, still to be verified, it is reasonable to assume that the reactions by patients to dextran in our formulation could be smaller than with Lymphoseek^®^. Further studies must be performed on this topic. 

#### 2.5.1. Stability Studies

The in vitro stability of ^99m^Tc-complexes was evaluated by monitoring radiochemical purity (RCP) at different time points (15, 30, 60, 120 min) by high-performance liquid chromatography (HPLC). After preparation, 100 µL of the selected radioactive compound were incubated at 37 °C with 900 µL of saline or, alternatively, rat serum. No significant variation of RCP was observed in both conditions.

#### 2.5.2. Cysteine and Glutathione (GSH) Challenge

An aliquot of freshly prepared aqueous solution of l-cysteine or GSH (50 µL, 10.0 mM) was placed in a test tube containing phosphate buffer (250 µL, 0.2 M, pH = 7.4), water (100 µL), and the appropriate ^99m^Tc-complex (100 µL). The mixture was incubated at 37 °C for 2 h. A blank experiment was carried out using an equal volume of saline. Aliquots of the resulting solutions were withdrawn at 15, 30, 60, 120 min after incubation and analyzed by HPLC chromatography. The complexes were found to be inert toward transchelation by cysteine and GSH.

## 3. Materials and Methods

### 3.1. General

Dextran with average molecular weight 10,000 Da, succinic dihydrazide [SDH=H_2_N–NH–(O=)C–(CH_2_)_2_–C(=O)–NH–NH_2_], sodium dihydrogen phosphate monohydrate (NaH_2_PO_4_·H_2_O), disodium hydrogen phosphate heptahydrate (Na_2_HPO_4_·7H_2_O), SnCl_2_·2H_2_O, tris(2-cyanoethyl)phosphine [PCN=P(CH_2_CH_2_CN)_3_], γ-hydroxypropylcyclodextrin, l-cysteine, and glutathione (GSH) were obtained from Sigma Aldrich, Milan, Italy.

Technetium-99m, as Na[^99m^TcO_4_] in physiological solution, was obtained from a Drytec™ ^99^Mo/^99m^Tc generator (GE Healthcare, Belfast, UK).

The infrared spectra (IR) (PerkinElmer, Waltham, Massachusetts, US) were recorded with FT-Perkin Elmer Spectrum 100 using a universal ATR crystal Zr/Se Diamond Bounces 1, serial number 14031; 1H-NMR spectra were recorded on a Varian 400 NMR instrument (Varian Inc., Palo Alto, CA, USA); the chemical shift (δ) is expressed in ppm.

#### 3.1.1. Synthesis of **1**

Sodium hydroxide (NaOH, 0.5 g, 12.5 mmol) and sodium borohydride (NaBH_4_, 20.0 mg, 0.53 mmol) were added to a stirred solution of dextran (average molecular weight = 10,000 Daltons) (2.0 g, 0.2 mmol) in water. Allyl bromide (BrCH_2_CH=CH_2_, 3.5 g, 30 mmol) was then added to this solution at 40 °C. The mixture was stirred at 60 °C for 3 h and then neutralized with acetic acid. The product was purified by repeated precipitation with ethanol and dried under vacuum to achieve a constant weight. The final loading of dextran polymer was achieved by comparison of ^1^H-NMR spectra. In particular, the chemical shift and the integral value of anomeric protons (nonsubstituted and substituted dextran) at 4.96 ppm and 5.13 ppm were reported to the allylic proton at 5.96 ppm. The loading was about 18 allylic groups for every polymeric unit (18 allyl moiety for 55 monomeric sugar in the dextran).

#### 3.1.2. Synthesis of Thioglycol Amide

Sodium azide (NaN_3_, 3.34 g, 51.38 mmol) was added to a stirred solution of 2-chloro-ethylamine hydrochloride (H_2_NCH_2_CH_2_Cl, 2.0 g, 17.39 mmol) in water, and the reaction solution was heated at 80 °C for 15 h. After cooling the reaction at 0 °C, KOH pellets were added until pH = 14. The aqueous solution was extracted 3 times with diethyl ether (30 mL each), and the resulting organic phase was separated, dried, and concentrated under vacuum to obtain the corresponding amino-azide compound H_2_NCH_2_CH_2_N_3_ (caution: explosive compound).

This compound was successively used to prepare the corresponding thioazide using the following procedure. The amino-azide (H_2_NCH_2_CH_2_N_3_, 0.33 g, 3.8 mmol), WSC (0.42 g, 2.19 mmol), and HOBt (0.18 g, 2.19 mmol) were added to a stirred solution of thioglicolic acid (0.33 g, 1.99 mmol) in DMF at 0 °C. The reaction mixture was stirred at room temperature for 24 h and then concentrated under vacuum and diluted with ethyl acetate. The organic phase was washed with a citric acid solution (10% in water, 30 mL), NaHCO_3_ (5% in water, 30 mL), and Brine (30 mL). The organic phase was dried and concentrated to dryness to obtain the thiozide.

^1^H NMR (400 MHz, Chloroform-*d*) δ 6.64 (bs, 1H), 3.59–3.54 (m, 1H), 3.36–3.31 (m, 4H), 3.29–3.23 (m, 2H).

^13^C NMR (100 MHz, Chloroform-*d*) δ 171.60, 49.84, 41.37, 32.75.

The allyl dextran (**1**) (80.0 mg) and the thioazide HSCH_2_C(=O)NHCH_2_CH_2_N_3_ (86.0 mg, 0.53 mmol) were dissolved in a mixture of water and THF(1:1). Ammonium persulfate (80.0 mg, 0.35 mmol) was added in one pot and the mixture was then heated at 50 °C for 2 h. After evaporation of the solvent under vacuum, the resulting product (**2**) was purified by gel filtration using a Sephadex G25 PD 10 column using water as eluent.

**Figure 12:**
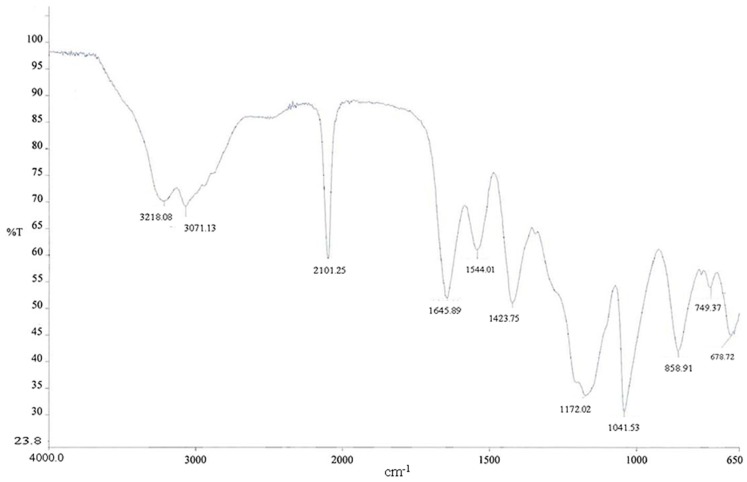
IR spectra of azido dextran after purification procedure.

As depicted in Figure 12, the infrared spectra of azido dextran showed the classical absorption peak at 2101 cm^−1^.

#### 3.1.3. Synthesis of **3**

The dextran moiety **3** was prepared according to literature methods [29]. The Fmoc-Cys-OEt group was attached to the allyl moiety using ammonium persulfate in water. In order to evaluate the derivatization loading of allyl-dextrane, we performed, in a small part of the product, a Fmoc deprotection and titration [30]; this analysis allowed us to determine the final loading of dextran in 0.20 mmol/g. The deprotection of Fmoc residue from the cysteine with DMF/20% piperidine yielded the free amine product **3**.

#### 3.1.4. Synthesis of **4**

To a stirred solution of HDTCZ (2.0 g, 14.68 mmol) in anhydrous THF (60 mL), DIPEA (2.84 g, 22.02 mmol) and propargyl chloroformate (2.61 g, 22.02 mmol) were added at 0 °C. The reaction was stirred at room temperature for 4 h and then quenched with a saturated solution of ammonium chloride to afford compound **4**.

^1^H NMR (400 MHz, Chloroform-*d*) δ 8.01 (bs, 1H), 4.85 (s, 2H), 2.61 (m, 4H).

#### 3.1.5. Synthesis of **5**

Compound **4** (5.3 mg, 3.69 × 10^−4^ mmol), sodium ascorbate (0.8 mg, 4.059 × 10^−5^ mmol), and CuSO_4_ (0.01 mg, 4.059 × 10^−6^ mmol) were added to a stirred solution of compound **2** (5.69 mg, approximately 4.06 × 10^−4^ mmol) in water (3.0 mL). The solution was stirred at room temperature for 12 h and then concentrated under vacuum to yield compound **5**.

#### 3.1.6. Synthesis of 2-(2,3,4,6-Tetra-*O*-acetyl-β-d-Mannopyranosyl)-Acetaldehyde (**7**)

A 0.5–2.0 mL process vial was filled with the α-mannosyl aldehyde **6** (150 mg, 0.40 mmol) and MeOH (1.5 mL). The resulting solution was cooled to 0 °C, and then l-proline (14 mg, 0.12 mmol) was added in one portion. The vial was sealed with the Teflon septum and aluminium crimp by using an appropriate crimping tool. The mixture was then vigorously stirred at 0 °C for 1 h, then the vial was placed in its correct position in the Biotage Initiator cavity where irradiation at constant power (13 W) was performed for 3 h with simultaneous cooling of the vial (internal temperature ≈60 °C) by means of pressurized air (4 bar). After the full irradiation sequence was completed, the vial was cooled to room temperature and then opened. The mixture was diluted with AcOEt (80 mL) and washed with saturated NaHCO_3_ (2 × 15 mL) and brine (2 × 5 mL). The organic phase was dried (Na_2_SO_4_), filtered, and concentrated to give crude β-mannosyl aldehyde **7** (β/α ratio 10:1). Flash column chromatography with 1:1 cyclohexane-AcOEt (containing 20% of CH_2_Cl_2_ and 1% of MeOH) afforded pure **7** (112 mg, 75%) as a white amorphous solid. ^1^H NMR: δ = 9.75 (dd, 1 H, *J* = 0.5 Hz, *J* = 1.5 Hz, CHO), 5.36 (dd, 1 H, *J* = 0.5 Hz, *J* = 3.0 Hz, H-2′), 5.24 (dd, 1 H, *J* = 9.0 Hz, *J* = 9.2 Hz, H-4′), 5.12 (dd, 1 H, *J* = 3.0 Hz, *J* = 9.2 Hz, H-3′), 4.30–4.20 and 4.18–4.06 (2 m, 3 H, H-1′, 2 h-6′), 3.72 (ddd, 1 H, *J* = 2.5 Hz, *J* = 6.0 Hz, *J* = 9.0 Hz, H-5′), 2.77 (ddd, 1 H, *J* = 1.5 Hz, *J* = 7.5 Hz, *J* = 17.0 Hz, H-2a), 2.54 (ddd, 1 h, *J* = 0.5 Hz, *J* = 4.5 Hz, *J* = 17.0 Hz, H-2b), 2.20, 2.10, 2.05, and 1.98 (4 s, 12 H, 4 Me). ESI MS (374): 397 (M + Na^+^).

#### 3.1.7. Synthesis of 2-(2,3,4,6-Tetra-*O*-Acetyl-β-d-Mannopyranosyl)-Acetic Acid (**8**)

A mixture of β-aldehyde **7** (112 mg, 0.30 mmol), sodium chlorite (271 mg, 3.00 mmol), sodium dihydrogen phosphate monohydrate (311 mg, 2.25 mmol), 2-methyl-2-butene (1.2 mL), *t*-BuOH (5.5 mL), and H_2_O (2.1 mL) was stirred at room temperature for 4 h and then diluted with CH_2_Cl_2_ (15 mL) and H_2_O (5 mL). The organic layer was separated and the aqueous layer was extracted with CH_2_Cl_2_ (3 × 10 mL) then acidified (pH 2) with 5% HCl and extracted again with CH_2_Cl_2_ (3 × 10 mL). The combined organic phases were dried (Na_2_SO_4_), filtered, and concentrated to give the β-mannosyl acetic acid **8** (111 mg, 95%) at least 95% pure as established by ^1^H NMR analysis. ^1^H NMR: δ = 5.40 (dd, 1 h, *J* = 0.5 Hz, *J* = 3.0 Hz, H-2′), 5.24 (dd, 1 H, *J* = 9.0 Hz, *J* = 9.2 Hz, H-4′), 5.11 (dd, 1 H, *J* = 3.0 Hz, *J* = 9.2 Hz, H-3′), 4.27 (dd, 1 H, *J* = 5.5 Hz, *J* = 12.0 Hz, H-6′a), 4.14 (ddd, 1 H, *J* = 0.5 Hz, *J* = 5.0 Hz, *J* = 7.5 Hz, H-1′), 4.10 (dd, 1 H, *J* = 3.0 Hz, *J* = 12.0 Hz, H-6′b), 3.70 (ddd, 1 H, *J* = 3.0 Hz, *J* = 5.5 Hz, *J* = 9.0 Hz, H-5′), 2.68 (dd, 1 H, *J* = 7.5 Hz, *J* = 17.0 Hz, H-2a), 2.50 (dd, 1 H, *J* = 5.0 Hz, *J* = 17.0 Hz, H-2b), 2.20, 2.08, 2.04, and 1.98 (4 s, 12 H, 4 Me). ESI MS (390): 413 (M + Na^+^).

#### 3.1.8. Synthesis of Pseudopeptide **9**

Fmoc-Rink amide resin (0.69 mmol/g, 0.2 g) was treated with piperidine [20% in *N*,*N*-dimethylformamide (DMF)] and linked with Fmoc-aa-OH (4.0 equiv) by using [*O*-(7-azabenzotriazol-1-yl)-1,1,3,3-tetramethyluronium hexafluorophosphate] (HATU, 4.0 equiv) as a coupling reagent. The coupling reaction was continued for 1 h and then piperidine (20% in DMF) was used to remove the Fmoc group at every step. The peptide resin was washed with methanol and dried in vacuum to yield the protected peptide-resin. This resin was treated with a mixture of trifluoroacetic acid (TFA)/H_2_O/Et_3_Si (9:0.5:0.5) for 1 h at room temperature. After filtration of the resin, the solvent was concentrated in vacuum and the residue triturated under diethyl ether. The crude linear peptide was purified by preparative reversed-phase HPLC to yield a white powder after lyophilization. Further purification was obtained by preparative reversed-phase HPLC using a Water Delta Prep 4000 system equipped with a Waters PrepLC 40-mm Assembly C18 column (30 × 4 cm, 300 A, 15 mm spherical particle size column). The column was perfused at a flow rate of 40 mL min^−1^ with solvent A (5% *v*/*v* acetonitrile in 0.1% aqueous TFA) and a linear gradient from 0 to 50% of solvent B (80% *v*/*v* acetonitrile in 0.1% aqueous TFA) over a period of 25 min. Analytical HPLC was performed on a Beckman 125 instrument fitted with an Alltech C18 column (4.6 × 150 mm, 5 mm particle size) and equipped with a Beckman 168 diode array detector. Analytical purity and retention time (t_R_) of **9** were determined using the solvent system A + B as specified above, at a flow rate of 1.0 mL min^−1^, and using a linear gradient ranging from 5 to 40% B over 25 min. Molecular weight of **9** was measured by ESI-MS analysis using a Micromass ZMD 2000 mass spectrometer.

HPLC: Rt 10.60 min; ESI MS (812): 813.3 (M + H^+^).

#### 3.1.9. Synthesis of Pseudopeptide **10**

Peptide **10** was obtained through a Fmoc-chemistry solid phase peptide synthesis using a Rink amide resin to elongate the peptide backbone starting from the C terminal (Cys). The pseudopeptide was cleaved from the resin using a mixture of TFA/water and triethylsilane.

HPLC: Rt 9.98 min; ESI MS (846): 847.6 (M + H^+^).

#### 3.1.10. Synthesis of **18**

**Figure 13:**
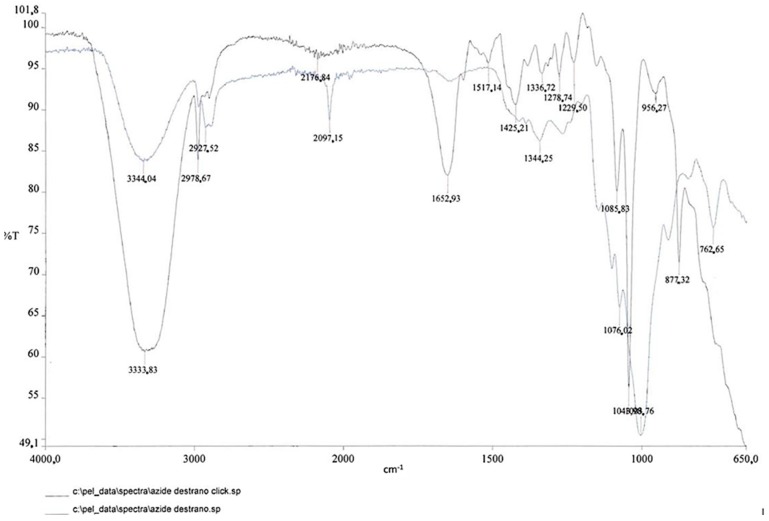
Comparison of IR spectra before and after click reaction.

To a stirred solution of compound **2** (5.69 mg, approximately 4.06 × 10^−4^ mmol) in water (3.0 mL) was added 9 (6.0 mg, 3.69 × 10^−4^ mmol), sodium ascorbate (0.8 mg, 4.059 × 10^−5^ mmol), and CuSO_4_ (0.01 mg, 4.059 × 10^−6^ mmol). The solution was stirred at room temperature for 12 h and then concentrated under vacuum to yield compound **18**. As depicted in Figure 13, the IR spectra showed the disappearance of azide peak (at 2097 cm^−1^) and the appearance of a new broad signal at 1652 cm^−1^ that could be assigned to carbonyl stretching of peptide amide moiety.

HPLC: Rt = 14.30 min.

#### 3.1.11. Synthesis of **19**

Using a classical peptide chemistry condensation, pseudopeptide **10** (6 mg, 0.00709 mmol) was condensed with dextran moiety **3** (40 mg, loading 0.2 mmol/g, 0.00788 mmol), using as coupling agents WSC (1.5 mg, 0.007799 mmol) and HOBt (1.2 mg, 0.007799 mmol).

HPLC: Rt = 14.30 min.

### 3.2. Preparation of ^99m^TcN-“3 + 1” Labeled Dextran-Mannose Derivate

Freshly generator-eluted Na[^99m^TcO_4_] (100 MBq, 0.9 mL) was added to a nitrogen-purged vial containing 1.0 mg of succinic dihydrazide (SDH) and 0.1 mg of SnCl_2_. The vial was kept at room temperature for 15 min to yield the [^99m^TcN]^2+^ group. The appropriate dextran-mannose derivate (0.1 mg dissolved in 0.5 mL of saline) and tris(2-cyanoethyl) phosphine (PCN, 0.5 mg dissolved in a saline solution containing 2.0 mg of γ-hydroxypropylcyclodextrin) were freshly prepared and then simultaneously added to the reaction vial containing the radioactive Tc-99m nitrido intermediate. The resulting mixture was heated at 80 °C for 15 min. The radiochemical yield, as determined by radio-HPLC chromatography, ranged from 95 to 98%.

### 3.3. Chromatography

The RCP of the final Tc-99m compounds was determined by HPLC performed on a Beckman System Gold Instrument equipped with a programmable solvent Module 126, scanning detector Module 166, and a radioisotope detector Module 170. Chromatographic analyses were carried out on a reversed-phase Agilent precolumn Zorbax 300SB-C18 (4.6 × 12.5 mm) and a reversed-phase Agilent column Zorbax 300SB-C18 (4.6 × 250 mm) using the following conditions. Mobile phase: A = water containing 0.1% TFA, B = acetonitrile containing 0.1% TFA; gradient: 0 min, B = 0%; 0–25 min, B = 100%; 25–30 min, B = 100%; 30–35 min, B = 0%; flow rate: 1.0 mL/min.

## 4. Conclusions

A new particular of mannosyl-dextran-derived multifunctional ligands, potentially useful for sentinel node detection, has been reported. These multifunctional ligands have been specifically designed to accommodate, in a controlled way, the same number of functional groups, each performing a specific chemical or biological function on the selected position on the dextran scaffold. Click reactions, as well as standard amide condensation, allowing the use of modular building blocks, have proven to be very efficient for building up the multifunctional ligands, containing a chelating system specifically chosen to label the [^99m^TcN]^2+^. We have found no differences between in vitro stability of the ^99m^TcN-“3+1” labeled dextran-mannose derivate obtained through the different chemical procedures. However, further in vivo investigations should be performed to confirm our results.
